# Structural and Functional Pulmonary Assessment in Severe COVID-19 Survivors at 12 Months after Discharge

**DOI:** 10.3390/tomography8050216

**Published:** 2022-10-13

**Authors:** Andrea Corsi, Anna Caroli, Pietro Andrea Bonaffini, Caterina Conti, Alberto Arrigoni, Elisa Mercanzin, Gianluca Imeri, Marisa Anelli, Maurizio Balbi, Marina Pace, Adriana Zanoletti, Milena Capelli, Fabiano Di Marco, Sandro Sironi

**Affiliations:** 1 Department of Radiology, ASST Papa Giovanni XXIII, 24127 Bergamo, Italy; 2 School of Medicine, University Milano Bicocca, 20126 Milan, Italy; 3 Bioengineering Department, Istituto di Ricerche Farmacologiche Mario Negri IRCCS, 24126 Bergamo, Italy; 4 Respiratory Unit, ASST Papa Giovanni XXIII, 24127 Bergamo, Italy; 5 Department of Health Sciences, University of Milan, 20146 Milan, Italy; 6 Unit of “Scienze Radiologiche”, Department of Medicine and Surgery, University of Parma, 43126 Parma, Italy; 7 Department of Radiology, ASST Bergamo EST, 24068 Bergamo, Italy

**Keywords:** COVID-19, lung diseases, respiratory function tests, tomography, severe acute respiratory syndrome coronavirus 2

## Abstract

Long-term pulmonary sequelae in COVID-19 patients are currently under investigation worldwide. Potential relationships between blood sampling and functional and radiological findings are crucial to guide the follow-up. In this study, we collected and evaluated clinical status, namely symptoms and patients’ reported outcome, pulmonary function tests (PFT), laboratory tests, and radiological findings at 3- and 12-months post-discharge in patients admitted between 25 February and 2 May 2020, and who survived severe COVID-19 pneumonia. A history of chronic pulmonary disease or COVID-19-unrelated complications were used as exclusion criteria. Unenhanced CTs were analyzed quantitatively (compromising lung volume %) and qualitatively, with main patterns of: ground-glass opacity (GGO), consolidation, and reticular configuration. Patients were subsequently divided into groups based on their radiological trends and according to the evolution in the percentage of compromised lung volume. At 12 months post-discharge, seventy-one patients showed significantly improved laboratory tests and PFT. Among them, 63 patients also underwent CT examination: all patients with negative CT findings at three months (*n* = 14) had negative CT also at 12 months; among the 49/63 patients presenting CT alterations at three months, 1/49 (2%) normalized, 40/49 (82%) improved, 7/49 (14%) remained stably abnormal, and 1/49 (2%) worsened. D-dimer values were low in patients with normal CT and higher in cases with improved or stably abnormal CT (median values 213 vs. 329 vs. 1000 ng/mL, respectively). The overall compromised lung volume was reduced compared with three months post-discharge (12.3 vs. 14.4%, *p* < 0.001). In stably abnormal CT, the main pulmonary pattern changed, showing a reduction in GGO and an increase in reticular configuration. To summarize, PFT are normal in most COVID-19 survivors 12 months post-discharge, but CT structural abnormalities persist (although sensibly improved over time) and are associated with higher D-dimer values.

## 1. Introduction

The still ongoing pandemic emergency caused by coronavirus disease 2019 (COVID-19) has affected more than 607 million people globally, with more than 6.4 million deaths recorded by the World Health Organization (WHO) as of 14 September, 2022 [1]. The disease primarily affects the respiratory system, with pneumonia as the most frequent serious manifestation of the infection, potentially requiring hospitalization and intensive care admission. Various COVID-19 complications have been reported, including acute respiratory distress syndrome (ARDS), thromboembolic manifestations, cardiac and cardiovascular alterations, encephalopathy, and superinfections such as aspergillosis [2,3,4].

Despite the significant number of recoveries from COVID-19 pneumonia during the acute phase of the disease, many patients still experience symptoms at 12 months, mainly fatigue, dyspnea, and musculoskeletal pain [5,6,7,8]. Additionally, long-term pulmonary consequences have been increasingly acknowledged with concern (i.e., *long COVID*). Many studies documented persisting pulmonary alterations in COVID-19 patients a few months after discharge [9,10,11,12,13,14,15]. Reported abnormalities range from pulmonary function impairment, especially in the carbon monoxide diffusing capacity (DLCO) and in the total lung capacity (TLC) [9,10,16], along with radiological evidence of residual lung involvement, such as ground-glass opacities (GGOs), reticulation, bronchiectasis, and volume loss [10,11,12,13,14,15].

Several one-year CT follow-up studies are now available and an overall improvement over time seems to emerge [5,6,10,17,18,19,20,21,22,23]. However, additional evidence and extended observation periods are required to determine whether the changes reported in the first months will completely resolve, whether some of them will be residual, or even worsen over time [24]. This could induce different patient follow-up strategies, possibly increasing the observation time and focusing on specific imaging, laboratory, and functional tests. Additionally, this might optimize the clinical management of COVID-19 patients over time [10] and even suggest the need for a new clinical-radiological model to rule out the potential formation of pulmonary fibrosis [11,13] early.

On this basis, our study aimed at gaining additional insight into the COVID-19 long-term pulmonary sequelae by: (*a*) assessing clinical, laboratory, functional, and radiological findings 12 months after discharge in a cohort of severe COVID-19 survivors; (*b*) exploring possible associations between these findings; and (*c*) investigating the evolution of the clinical/functional and radiological patterns at 3- and 12-months post-discharge.

## 2. Materials and Methods

This retrospective observational study (*protocol Surviving COVID-19*) was approved by the local Institutional Review Board (Comitato Etico di Bergamo, Bergamo, Italy), which waived the need for written informed consent due to the pandemic contingency.

### 2.1. Study Population and Data Collection

The study cohort includes 71 severe COVID-19 patients admitted to Papa Giovanni XXIII Hospital (Bergamo, Italy) between 25 February and 2 May 2020). These survivors underwent clinical and pulmonary function evaluation and chest CT three months after being discharged, as previously described in [14]. The total population was followed up at 12 months after discharge with clinical and pulmonary function assessment, while 63/71 patients also had chest CT scans.

The inclusion criteria were: (*a*) COVID-19 diagnosis labeled as severe according to WHO interim guidance [25], (*b*) chest radiograph acquired at the time of the diagnosis, (*c*) pulmonary function tests (PFT) at three and 12 months after discharge, and (*d*) chest CT data at three and, whenever available, 12 months. The exclusion criteria were: (*a*) history of chronic pulmonary disease of any cause, (*b*) PFT that were not considered acceptable when not compliant with ATS/ERS 2019 standards [26], (*c*) low-quality CT (motion/respiratory artifacts impairing image quality), and (*d*) post-discharge COVID-19 unrelated diseases onset.

Additional descriptive and clinical information was extracted from the electronic records: demographic data, smoking history, comorbidities, and pulmonary assessment report (symptoms, laboratory data, namely D-dimer, CRP, WBC with lymphocytes and neutrophils sub-analysis, and PFT) at three and 12 months after discharge. Questionnaires commonly used for other respiratory diseases, such as chronic obstructive pulmonary disease (COPD), were administered 12 months after discharge (namely, the EQ-5D health-related quality of life questionnaire [27], the hospital anxiety and depression scale—HADS [28], and the St. George’s Respiratory Questionnaire—SGRQ [29]).

### 2.2. CT Image Acquisition, Qualitative and Quantitative Analysis

Chest CTs were performed unenhanced, in the supine position and full inspiration, from the lung bases to the apex, with either a 64- or a 16-slice scanner (Brilliance 64 and MX 16-slice; Philips Medical Systems, Best, The Netherlands): the acquisition parameters have been previously detailed [14].

CT qualitative evaluations were performed by three radiology residents (M.B. for the CTs performed at three months; A.C. and E.M. for the CTs performed at 12 months), and all readings of the residents were re-evaluated by a radiologist with 10 years’ experience (P.A.B.), all blinded to clinical data other than COVID-19 history. CT findings description relied on the *Fleischner Society* terminology and conformed with the peer-reviewed literature on viral pneumonia [30,31]. For each scan, the reviewers visually detected the prevailing pattern: ground-glass opacity (GGO), consolidation, reticular configuration (covering reticulation, parenchymal bands, or subpleural curvilinear opacity without substantial GGO or consolidation). In case no patterns prevailed, the most representative couple was reported. Additional assessments addressed the existence of bronchial dilatation, in contrast to bronchiectasis, architectural distortion, cavitation, and pleural effusion. Pulmonary lobes involved were recorded. CT alterations were furtherly classified by topography in (*a*) peripheral (mainly peripheral one-third of the lung), central (central two-thirds of the lung), or neither; (*b*) unilateral/bilateral; and (*c*) predominantly upper (above the carina), middle (between the carina and the right inferior pulmonary vein), lower (under the right inferior pulmonary vein), or none.

According to the radiological trend over 12 months after discharge, patients with 12-month CT follow-up (63/71) were retrospectively divided into five groups: chest CT already normal at three months (*group 1*); abnormal chest CT at three months which became normal at 12 (*group 2*); chest CT still abnormal at 12 months, but improved (*group 3*), stably abnormal (*group 4*) or worsened (*group 5*) compared to 3-month CT.

Abnormal lung volume quantification was performed using the open-source 3D Slicer software, version 4.8.1 (https://www.slicer.org accessed on 11 October 2022) [32], as previously described [7]. The procedure included lung segmentation and airways exclusion, performed using the Chest Imaging Platform extension (Applied Chest Imaging Laboratory, Boston, MA, USA) and the Airway Segmentation Module. A −950 HU baseline minimum was imposed, and lung regions were classified as altered parenchyma for image density over −800 HU. Manual editing of the result accounted for inaccuracies.

### 2.3. Post-Discharge Pulmonary Assessment

The post-discharge pulmonary assessment included PFT, laboratory tests, and symptoms assessment.

PFT was performed by professionally trained respiratory technicians based on the current standards [26] and employing Medical Graphics Elite Pro body box equipped with rapid gas analyzers (MGC Diagnostics Corporation, St. Paul, MN, USA). PFT results were interpreted by two experienced pulmonologists (C.C. and G.I.) per current guidelines [33]. Spirometric parameters included: forced vital capacity (FVC), forced expiratory volume in the 1st second (FEV1), FEV1/FVC ratio, alveolar volume (VA), diffusing capacity for carbon monoxide (DLCO), carbon monoxide transfer coefficient (KCO), and—at 12-month after discharge only—total lung capacity (TLC). PFT parameters were expressed as a percentage of the predicted value and considered impaired when lower than the lower limit of the normal range (LLN) defined by the Global Lung Function Initiative 2012 reference equations for spirometry [34] and the Global Lung Function Initiative 2017 reference equations for DLCO [35]. TLC results were expressed as a percent of the predicted value based on ECSC reference equations and considered impaired when lower than the LLN [36]. The dyspnea intensity was assessed using the modified Medical Research Council (mMRC) dyspnea scale.

### 2.4. Statistical Analysis

At 12 months after discharge, clinical and laboratory findings in patients with normal vs. abnormal chest CT were compared by Mann–Whitney or Fisher tests (numerical and binary/categorical variables, respectively). Clinical, laboratory and radiological data at 12 months were compared with paired data at three months after discharge by paired *t*-test (continuous variables with normal distribution), paired Mann–Whitney test (continuous variables with non-normal distribution), McNemar Chi-squared test (binary variables), Dependent-samples Sign-Test (ordinal variables—mMRC), or marginal homogeneity test (categorical variables). Overall and pairwise differences in DLCO percentage of the predicted value and D-dimer values by the radiological trend of the 12 months after discharge were assessed by the Kruskal–Wallis and Wilcoxon tests, respectively. Statistical significance was set at *p* < 0.05. All statistical analyses were performed using R software (R Core Team, Vienna, Austria), version 3.6.3.

## 3. Results

### 3.1. Patient Population

**Figure 1:**
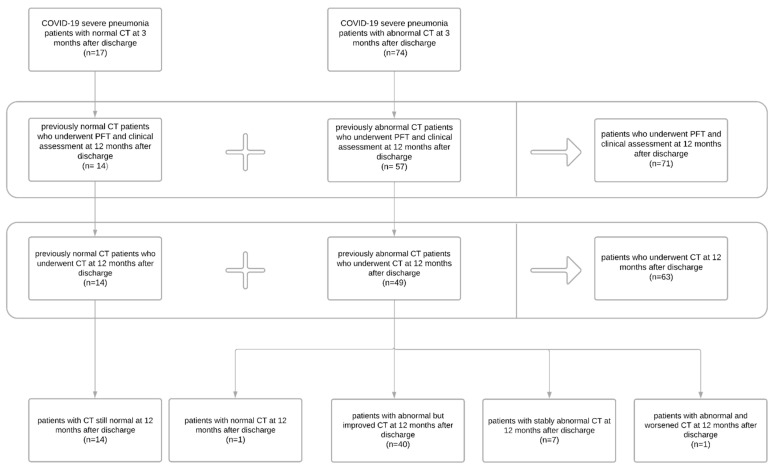
Flow chart of the study participants.

Ninety-one severe COVID-19 survivors with clinical assessment, PFT, and chest CT at 3 months after discharge, already participating in a previous study [14], were considered for inclusion in the current study (Figure 1): 17/91 had normal CT three months after discharge, 74/91 had abnormal CT. Among the 17 patients who had normal CT at three months, 14/17 underwent PFT and clinical assessment alongside CT scan 12 months after discharge; 3 patients from this group were lost at follow-up. Among the 74 patients who had abnormal CT 3 months after discharge, 57/74 underwent PFT and clinical assessment, and only 49/74 underwent CT 12 months after discharge; 17 patients from this group were lost at follow-up.

Therefore, a total of 71 patients with PFT and clinical assessment at 12 months was finally included in the current study (26 females [37%]; median age 66 years, IQR = [59–73]). Demographic and clinical information of the study population is reported in Table 1.

### 3.2. Clinical Assessment, Laboratory Data, and Pulmonary Function

In comparison to the laboratory findings at three months after discharge, at 12 months patients showed lower concentrations of white blood cells (6.24 vs. 6.55 × 10^9^/L; *p* = 0.029), lymphocytes (2.04 vs. 2.10 × 10^9^/L; *p* = 0.022) and neutrophils (3.19 vs. 3.61 × 10^9^/L; *p* = 0.021). Symptoms remained almost unchanged between three and 12 months, except for sputum, for which the prevalence increased from 3% to 13% (Table 2).

PFT results significantly improved from 3 to 12 months, with an increase in FVC (97 vs. 89%, *p* < 0.001), FEV1 (97 vs. 91%, *p* < 0.001), DLCO (95 vs. 85%, *p* < 0.001), and KCO percentage of the predicted value (101 vs. 95%, *p* < 0.001). Therefore, the prevalence of pulmonary function abnormalities showed a significant reduction, with only 4% of patients with FVC%, FEV1%, and KCO% and 7% of patients with DLCO% lower than the limit of normality 12 months after discharge (Table 2). However, at 12 months after discharge, the TLC percentage of the predicted value was still below the lower limit of normality in 12% of patients.

### 3.3. Patients’ Reported Outcomes

The St. George’s Respiratory Questionnaire scores at 12 months after discharge were significantly higher than normative values previously reported in a healthy population with no history of respiratory disease [37], both in terms of the total score (21 vs. 6) and each specific item, especially the activity rate (48 vs. 9). HADS questionnaire showed median values of anxiety and depression well under the abnormality threshold (Table 2), with 20% and 9% of patients showing values of 7 or more (borderline abnormality threshold) and only 3% and 3% values higher than 10 (abnormality threshold) for anxiety and depression respectively. The most impaired EQ-5D dimension was “pain”, with only 57% having none, while the EQ VAS showed normal values of overall health status taking into consideration the prevailing sex and median age of the examined population [38] (Table 2).

### 3.4. Chest CT Findings and Quantitative Analysis Results

A subgroup of patients (63/71) also underwent a chest CT scan 12 months after discharge. The ones lost at CT scan follow-up belonged to the normal and abnormal group at three months. In all 14/63 cases (22%) with negative chest CTs at three months, CT findings remained negative. Among 49/63 patients (78%), only in one case was the CT scan normalized at 12 months. In the remaining cases, CT findings, compared to three months post-discharge, improved but were still abnormal in 40/49 (82%) patients and remained almost unchanged in 7/49 (14%). Only one patient worsened (Figure 1), showing an increase in the reticular pattern.

**Figure 2:**
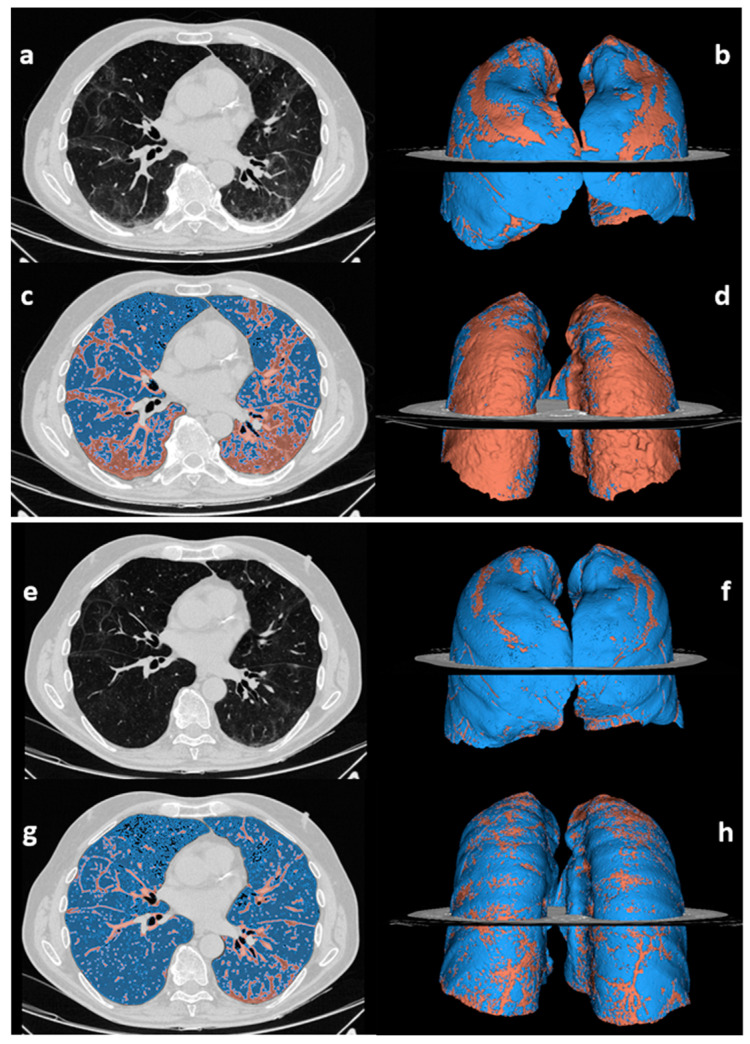
Abnormal lung volume quantification on chest CT scans acquired at 3 months (**a**–**d**) and 12 months after discharge (**e**–**h**) in a 70-year-old male patient who suffered from severe COVID-19. The unenhanced chest CT scans (**a**,**e**) are displayed alongside the pertinent segmentations of residual lung abnormalities (red) and normally aerated lung (blue) performed by 3D Slicer software (**c**,**g**), and the related 3D anterior (**b**,**f**) and posterior (**d**,**h**) volumetric representations. The case is representative of the subgroup of patients with improved but still abnormal chest CT findings (*group 3*). The percentage of abnormal lung volume decreased over time, from 33% at three months (**b**,**d**) to 13% at 12 months after discharge (**f**,**h**). The dorsal segments are the most impacted by residual CT findings at 12 months.

At 12 vs. three months post-discharge, the percentage of compromised lung volume reduced (12.3 vs. 14.4%, *p* < 0.001), as listed in Table 3 and visually exemplified in Figure 2.

**Figure 3:**
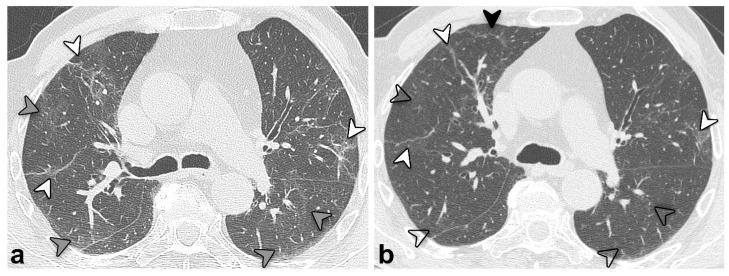
Unenhanced chest CT scan axial images of the same patient in Figure 2 (*group 3*) at 3 (**a**) and 12 months (**b**) after discharge. An overall improvement is evident, with previous GGO (grey arrowheads, (**a**)) almost resolved but showing a “*melting sugar*” appearance on the latter (empty arrowheads, (**b**)). Reticular opacities (white arrowheads, (**a**,**b**)) also improved but remained evident; the black arrowhead (**b**) highlights a new onset of tiny peripheral reticular opacification.

Moreover, the CT pattern significantly changed over time (*p* = 0.038; Table 3 and Figure 3), with a trend of reduction of GGO prevalence (4% at 12 vs. 16% at three months) and an increase in reticulation prevalence (79% at 12 vs. 63% at three months).

Persistent architectural distortion, bronchial dilation, and predominant peripheral axial distribution were noted, with no significant differences among the two CT sets. Cranio-caudal distribution turned statistically significant (*p* = 0.047; Table 3). Table 4 shows radiological findings over time in patients with improved (*group 3*) vs. stably abnormal (*group 4*) chest CT.

### 3.5. Structural and Functional Findings’ Association

Patients with chest CT still abnormal at 12 months after discharge (*n* = 7, *group 4*) had lower DLCO (95 vs. 102%, *p* = 0.085) and a significantly lower TLC percentage of the predicted value (95 vs. 105, *p* = 0.005), as compared with patients with normal chest CT (*n* = 14, *group 1*). On the contrary, the two groups’ clinical symptoms and laboratory findings were normal (Table 5).

**Figure 4:**
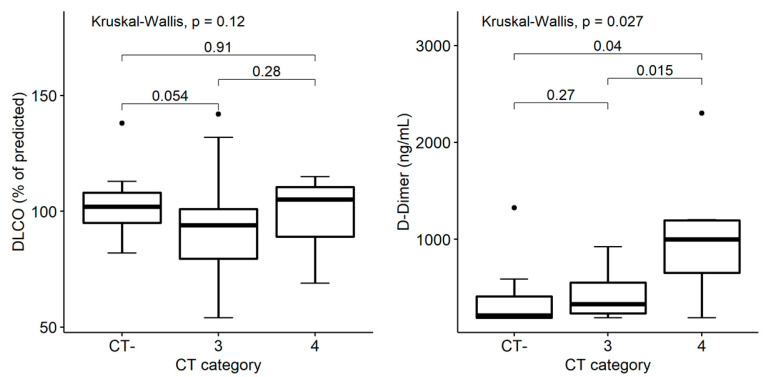
Association between radiological trend and clinical and laboratory findings 12 months after discharge among COVID-19 survivors, with normal (*CT-*), improved (*group 3*) and stably abnormal (*group 4*) chest CT findings. Box plots on the left show distribution of DLCO percentage of the predicted value by chest CT category. Box plots on the right show distribution of D-dimer values by chest CT category. *p*-values denote significance in overall and pairwise differences assessed by Kruskal–Wallis and Wilcoxon tests, respectively.

The D-dimer value was lowest in patients with a normal chest CT scan (*n* = 14, *group 1*), numerically higher in patients with improved CT findings (*n* = 40, *group 3*), and significantly higher in patients with stably abnormal chest CT at 12 months (*n* = 7, *group 4*): median values 213 vs. 329 vs. 1000 ng/mL, respectively (Kruskal–Wallis *p* = 0.027; Figure 4 left). On the contrary, the DLCO percentage of the predicted value was not different among the three groups of patients (*p* = 0.12; Figure 4 right)

## 4. Discussion

The present study’s findings show the normalization of pulmonary function tests’ results in most COVID-19 survivors at 12 months after discharge, along with the simultaneous reduction of structural abnormalities on chest CT assessed through a dedicated quantification analysis. Residual abnormalities at one year have been described in a variable percentage of patients, ranging from 20 to 56% [10,17,18,19,21]. An association between persistent CT abnormalities and the initial severity of the disease is currently suspected [7,21]. This may explain why, in our cohort, 78% of patients still showed morphologic lung abnormalities. Indeed, our population is composed of patients infected by the original strain during the first COVID-19 wave, when a severe disease at admission was common in Lombardy [39]. The same applies to other studies [13,14], with emerging evidence of a possible association between persistent alterations and ventilation supports [20]. Evidence also reported a progressive reduction in GGO while relative stability, or slow regression, of the reticular pattern [5,15,18,19,21,22], with only a few cases describing GGO as the predominant residual feature [10,20]. Other studies also documented an improvement in the ratio of lung zones that normalized during follow-up [5,19]. Our study is in line with these findings (compromised of lung % improvement), even if the method employed for assessing the extension of lung involvement is different (volume quantification vs. visual % evaluation of 6 lung zones or lobes). Residual architectural distortion and bronchiectasis were identified and also reported in other works [7,15,17,18,20,21]. While the first may be interpreted as scars, the latter has been found to be reversible [15]. In fact, residual CT abnormalities seem to improve over time. Fibrotic-like changes (e.g., parenchymal bands) may be due to limited areas of organizing pneumonia, while reducing GGO may be the expression of inflammation and immature fibrosis remodeling [15].

The main symptoms still present after one year were dyspnea and musculoskeletal pain, which are found to be the main ones also in the literature, along with fatigue [5,6,7,8], and regardless of the severity of the disease. Moreover, DLCO proved to correlate inversely with residual lung abnormalities [20,21].

We found no signs of depression, anxiety, or overall impairment on QoL in our patients, even if our sample showed a higher prevalence of EQ-5D dimensions impairment, especially the “pain” one, with 73% of patients having moderate or severe pain. Unfortunately, no PRO data were available at three months; thus, it is not possible to evaluate if symptoms improved over time. However, when compared with other studies that assessed SGRQ at 6 weeks after discharge, our patients show overall better scores, especially when considering symptoms and impact factors [40]. Other studies (e.g., Marando et al. and Gamberini et al. [5,6]) describe that HRQoL impairment is frequent one year after severe COVID-19, and the lowest recovery is found in the mental component.

On the contrary, the persisting structural abnormalities at 12 months and the change in CT pattern from 3 to 12 months after discharge highlight the importance of chest CT in monitoring COVID-19 pulmonary sequelae. The aforementioned post-discharge pattern change observed in the current study over the months (denoted by a reduction of GGO and increase in reticulation prevalence) has been previously described as a “tinted” or “melting sugar” sign. This does refer to a slightly increased extension and an attenuation of GGO and consolidations [41,42], likely indicative of gradual regression of the inflammation and re-expansion of the alveoli [12], as confirmed by the overall improvement in pulmonary function assessed in both spirometry and DLCO. Indeed, while patients with qualitatively improved chest CTs showed decreased compromised lung percentages, patients with stable but still abnormal CT showed a slightly increased extension, suggesting the need for combining qualitative and quantitative evaluations to assess lung structural damage reliably.

Abnormal D-dimer values may persist over time, as described by Sonnweber et al., who found high D-dimer values in 27% of COVID-19 patients at 100 days of follow-up, alongside increased NT-proBNP and serum ferritin [43]. In our study, D-dimer values were associated with chest CT findings and radiological trends. Patients with normal chest CTs had the lowest D-dimer values, while patients with stably abnormal imaging findings had higher D-dimer values than those with improved CTs. This suggests that, alongside imaging, D-dimer may help monitor COVID-19 long-term pulmonary complications, potentially identifying patients with persisting lung damage that would benefit the most from repeated chest CT scans. Current findings are in keeping with previous COVID-19 studies highlighting the potential of D-dimer as a possible predictor of future impaired pulmonary function, already from hospital admission [16]. D-dimer has also been reported as a possible sign and prognostic indicator of fibrotic-type changes, i.e., persistent traction bronchiectasis, parenchymal bands, and/or honeycombing [10,12].

Some limitations have to be addressed in the present study. First of all, a limited sample size. Furthermore, CT data from a few cases were missing, either because patients refused a new scan or because the exam was performed in an outside Institution. However, all these cases underwent PFT and clinical evaluation 12 months after discharge. Abnormal lung volume quantification, based on thresholding, can be affected by the image quality and noise and may be unable to detect minimal changes, resulting in a relatively stable percentage of compromised lung volume (Table 3) both in the improved and stably abnormal group (Table 4); this is also the main reason why all scans not performed in our Institution were excluded. Other limitations include: the lack of patient-reported outcomes at three months after discharge; the inability due to the study’s retrospective nature to access data regarding acute-phase medications (such as corticosteroids) and mechanical ventilation, found to potentially influence the course of COVID-19 sequelae [44,45,46,47]; the lack of CT scans acquired during the acute phase of the illness, and pre-COVID structural and functional findings. Also, data on static lung volumes at three months are missing due to the limitations caused by the post-pandemic contingency, making it impossible to evaluate any change in TLC in our sample. Given the variability across patients, future studies using the extreme value theory to investigate the extreme cases further could be of value.

## 5. Conclusions

COVID-19 lung involvement tends to decrease over time with a significant reduction in GGO at 12 months, while a slower resolution of fibrotic-type changes makes them the prevalent pattern in some cases. Although PFT almost normalizes, symptoms like dyspnea, fatigue, and musculoskeletal pain are still present after one year, thus not allowing to rule out long-term COVID-19 pulmonary complications. On the contrary, our findings show that D-dimer may act as a biomarker in predicting COVID-related sequelae, being associated with stably abnormal imaging findings. At least in these cases, follow-up, inclusive of an additional CT scan at 24–36 months, should be considered in order to understand further the clinical, functional, and radiological trend of COVID-19 lung sequelae.

## Figures and Tables

**Table 1:** Demographic and clinical features of the 71 patients who survived severe COVID-19 pneumonia [25] and were included in the study and followed up to 12 months after discharge.

No.	71
Age (years, range)	66 [59–73]
Sex, F	26 (37)
Smoking history (never/former/current)	33 (46)/38 (54)/0 (0)
**Comorbidities**	
Any	57 (81)
>2	19 (27)
Arterial hypertension	42 (59)
Cardiovascular disease *	14 (20)
Obesity **	20 (28)
Diabetes	12 (17)
Dyslipidemia	24 (34)
Chronic renal failure	1 (1)
Neoplasia (active history)	1 (1)
Rheumatic pathology	5 (7)
Immunodepression	3 (4)
Epilepsy	1 (1)
Cirrhosis	0 (0)

Data are reported as median [IQR] (continuous/numerical variables) or number (%) (binary/categorical variables). * Including coronary heart disease, cerebrovascular disease, heart failure, and peripheral vascular disease. ** Defined as BMI ≥ 30.

**Table 2:** Clinical and laboratory findings and patients’ reported outcomes in 71 COVID-19 survivors who underwent repeated clinical examinations up to 12 months after discharge.

	3-Month FU	12-Month FU	*p*
Follow-up duration *, days	105 [90–129]	393 [379–409]	
***Laboratory data***			
D-dimer (ng/mL) **	411 [244–749]	335 [209–583]	0.103
CRP (mg/dL)	0.2 [0.1–0.4]	---	---
WBC (10^9^/L)	6.55 [5.62–7.98]	6.24 [5.27–7.57]	0.029
Lymphocytes (WBC %)	32.9 [28.2–39.3]	32.6 [27.8–38.4]	0.498
Lymphocytes (10^9^/L)	2.10 [1.71–2.82]	2.04 [1.58–2.54]	0.022
<1—no./total no. (%)	4/69 (6)	6/71 (8)	0.480
Neutrophils (WBC %)	55.5 [48.1–61.8]	54.3 [48.5–59.4]	0.195
Neutrophils (10^9^/L)	3.61 [2.94–4.48]	3.19 [2.49–4.37]	0.021
***Symptoms***			
Asthenia	31 (44)	23 (32)	0.201
Dyspnea	48 (68)	41 (58)	0.230
mMRC dyspnea scale (0/1/2/3), no. (%)	19 (27)/40 (56)/12 (17)/0 (0)	25 (35)/33 (46)/10 (14)/3 (4)	0.500
Chest pain	4 (6)	4 (6)	1.000
Cough	10 (14)	13 (18)	0.579
Sputum	2 (3)	9 (13)	0.046
***Patients’ reported*** ***outcomes***			
EQ5D—Mobility (1/2/3)	---	48 (70)/21 (30)/0 (0)	---
EQ5D—Self-care (1/2/3)	---	65 (93)/4 (6)/1 (1)	---
EQ5D—Activity(1/2/3)	---	50 (71)/20 (29)/0 (0)	---
EQ5D—Pain (1/2/3)	---	39 (57)/27 (39)3 (4)	---
EQ5D—Anxiety (1/2/3)	---	51 (73)/17 (24)/2 (3)	---
EQ5D—Visual Analog Scale	---	80 [68–88]	---
HADS—Anxiety	---	3 [1–5]	---
>10—no./total no. (%)		2/66 (3)	
HADS—Depression	---	2 [1–4]	---
>10—no./total no. (%)		2/66 (3)	
SGRQ—Symptoms	---	18.27 [7.80–35.34]	---
SGRQ—Activity	---	47.58 [18.48–59.46]	---
SGRQ—Impact	---	8.85 [0.00–20.86]	---
SGRQ—Total	---	21.46 [11.34–33.90]	---
***Pulmonary function tests***			
FVC % predicted	89 [79–99]	97 [86–106]	<0.001
<LLN—no. (%)	11 (15)	3 (4)	0.027
FEV1 % predicted	91 [83–102]	97 [88–107]	<0.001
<LLN—no. (%)	10 (14)	3 (4)	0.046
FEV1/FVC % predicted	104 [99–108]	100 [97–105]	<0.001
<LLN—no. (%)	3 (4)	2 (3)	1.000
VA % predicted	92 [85–102]	95 [84–103]	0.079
<LLN—no. (%)	14 (20)	7/68 (10)	0.077
DLCO % predicted	85 [71–104]	95 [82–105]	<0.001
<LLN—no. (%)	24 (34)	5/68 (7)	<0.001
KCO % predicted	95 [81–103]	101 [87–112]	<0.001
<LLN—no. (%)	11 (15)	3/68 (4)	0.013
TLC % predicted	---	96 [83–104]	---
<LLN—no. (%)	---	8/69 (12)	---

Data are reported as median [IQR] (continuous/numerical variables) or number (%) (binary/categorical variables). *p*-values are computed by paired *t*-test (continuous variables with normal distribution), paired Mann–Whitney test (continuous variables with non-normal distribution), McNemar Chi-squared test (binary variables), or Dependent-samples Sign-Test (ordinal variables—mMRC). * From disease onset to CT date (or clinical visit in case of no CT). ** *n* = 5 and *n* = 10 missing data (FU1 and FU2, respectively). No patients’ reported outcomes were available at FU1. EQ5D items’ scores range from 1 to 3 (1 = no problem, 2 = moderate problem, 3 = severe problem), and the visual analogue scale (VAS) expresses the general health status (from 0 indicating the worst to 100 indicating the best health state). Abbreviations: CRP = C-reactive protein, DLCO = diffusion capacity for carbon monoxide, FEV1 = forced expiratory volume in the first second, EQ5D = European quality of life index version 5D; FVC = forced vital capacity, HADS = Hospital Anxiety and Depression Scale, KCO = carbon monoxide transfer coefficient, LLN = lower limit of normal, mMRC = modified Medical Research Council, SGRQ = St. George’s Respiratory Questionnaire, TLC = total lung capacity, VA = alveolar volume, WBC = white blood cells.

**Table 3:** Radiological findings in the COVID-19 survivors who underwent CT examination at three and 12 months after discharge and showed persistent chest CT abnormalities. Subclassification in improved/stably abnormal/worsened (group 3/4/5) is not considered in this summary.

	3-Month FU	12-Month FU	*p*
No. of abnormal chest CT scans	49/63 (78%)	48/63 (76%) *	1.000
% of compromised lung volume	14.4 [10.6–21.2]	12.3 [9.2–15.9]	<0.001
***Type of CT pattern***			0.038
GGO	8 (16)	2 (4)	
Reticular	31 (63)	38 (79)	
Combined	10 (21)	8 (17)	
Consolidation	2 (4)	2 (4)	1.000
Architectural distortion	45 (92)	46 (96)	1.000
Bronchial dilatation	44 (90)	42 (88)	0.683
Emphysema	7 (14)	9 (19)	0.617
Cavitation	1 (2)	0 (0)	NA **
Pleural effusion	1 (2)	1 (2)	1.000
Distribution			
Bilateral	47 (96)	48 (100)	NA **
Axial distribution (central/peripheral/neither)	1 (2)/18 (37)/30 (61)	0 (0)/28 (58)/20 (42)	NA **
Cranio-caudal distribution (superior/medium/inferior/none)	2 (4)/1 (2)/7 (14)/39 (80)	1 (2)/6 (13)/12 (25)/29 (60)	0.047
RUL	48 (98)	45 (94)	0.479
ML	46 (94)	45 (94)	1.000
RLL	46 (94)	46 (96)	1.000
LUL	43 (88)	40 (83)	0.617
Lingula	44 (90)	43 (90)	1.000
LLL	46 (94)	47 (98)	0.480

Data are reported as median [IQR] (continuous/numerical variables) or number (%) (binary/categorical variables). *p*-values were computed by paired *t*-test (continuous variables with normal distribution), paired Mann–Whitney test (continuous variables with non-normal distribution), McNemar Chi-squared test (binary variables), or Marginal homogeneity test (categorical variables). * Among 49 patients with CT abnormalities at three months, only one case became normal at 12 months reassessment. ** *p*-values could not be computed due to the different number of levels. Abbreviations: GGO = ground-glass opacity, RUL = right upper lobe, ML = middle lobe, RLL = right lower lobe, LUL = left upper lobe, LLL = left lower lobe.

**Table 4:** Radiological findings in 47 COVID-19 survivors * with an abnormal chest CT scan at 12 months after discharge, divided into improved (*group 3*) vs. stably abnormal (*group 4*), based on radiological finding trend (as described in the Section 2.2).

	Improved (*Group 3*), *n* = 40/47		Stably Abnormal (*Group 4*), *n* = 7/47	
	3 Months	12 Months	3 Months	12 Months
Compromised lung volume %	15.6 [11.0–21.7]	12.0 [9.2–14.3]	12.1 [10.9–20.5]	16.6 [11.8–19.7]
Type of CT pattern				
GGO	6 (15)	2 (5)	1 (14)	0 (0)
Reticular	28 (70)	34 (85)	2 (29)	3 (43)
Combined GGO- reticular	6 (15)	4 (10)	4 (57)	4 (57)
Consolidation	2 (5)	2 (5)	0 (0)	0 (0)
Architectural distortion	38 (95)	38 (95)	6 (86)	7 (100)
Bronchial dilatation	38 (95)	35 (88)	5 (71)	6 (86)
Emphysema	5 (13)	6 (15)	2 (29)	3 (43)
Cavitation	1 (3)	0 (0)	0 (0)	0 (0)
Pleural effusion	1 (3)	1 (3)	0 (0)	0 (0)
Distribution				
Bilateral	39 (98)	40 (100)	6 (86)	7 (100)
Axial distribution (central/peripheral/ neither)	1 (3)/13 (32)/26 (65)	0 (9)/24 (60)/16 (49)	0 (0)/4 (57)/3 (43)	0 (0)/3 (43)/4 (57)
Cranio-caudal distribution (superior/medium/inferior/none)	1 (3)/0 (9)/7 (17)/32 (80)	1 (3)/5 (12)/11 (2723(58)	1 (14)/1 (14)/0 (0)/5 (72)	0 (0)/1 (14)/1 (14)/5 (72)
RUL	40 (100)	38 (95)	6 (86)	6 (86)
ML	38 (95)	37 (93)	6 (86)	7 (100)
RLL	39 (98)	39 (98)	5 (71)	6 (86)
LUL	36 (90)	33 (83)	5 (71)	6 (86)
Lingula	37 (93)	33 (90)	6 (86)	6 (86)
LLL	39 (98)	40 (100)	5 (71)	6 (86)

Data are reported as median [IQR] (continuous/numerical variables) or number (%) (binary/categorical variables). * Among 49 patients with CT abnormalities at three months, one case became normal at 12 months reassessment (*group 1*), and 1 worsened (*group 5*). Abbreviations: GGO = ground-glass opacity, RUL = right upper lobe, ML = middle lobe, RLL = right lower lobe, LUL = left upper lobe, LLL = left lower lobe.

**Table 5:** Clinical and laboratory findings at 12 months after discharge in 63 COVID-19 survivors, subdivided by chest CT findings at 12 months: normal (CT-) vs. abnormal (CT+).

	CT-	CT+	*p*
*n*	15	48	
***Laboratory data***			
D-dimer (ng/mL) **	213 [190–410]	446 [260–596]	0.136
WBC (10^9^/L)	5.50 [4.52–6.63]	6.27 [5.37–7.57]	0.162
Lymphocytes (WBC %)	32.9 [31.0–39.5]	32.0 [25.4–38.4]	0.303
Lymphocytes (10^9^/L)	2.06 [1.68–2.56]	2.03 [1.47–2.53]	0.810
<1—no./total no. (%)	1/14 (7)	4/47 (9)	1.000
Neutrophils (WBC %)	52.9 [49.0–57.5]	56.1 [47.7–62.3]	0.451
Neutrophils (10^9^/L)	2.89 [2.36–3.17]	3.34 [2.55–4.33]	0.107
***Symptoms***			
Asthenia	3 (21)	16 (34)	0.516
Dyspnea	10 (71)	25 (53)	0.356
mMRC dyspnea scale (0/1/2/3), no. (%)	4 (29)/6 (43)/3 (21)/1 (7)	18 (38)/22 (47)/5 (11)/2 (4)	0.564
Chest pain	1 (7)	2 (4)	0.550
Cough	4 (29)	7 (14)	0.256
Sputum	2/13 (15)	5 (10)	0.639
***Pulmonary function tests***			
FVC % predicted	102 [91–111]	96 [86–107]	0.359
<LLN—no. (%)	1/14 (7)	0/47 (0)	0.230
FEV1 % predicted	100 [92–110]	97 [88–107]	0.619
<LLN—no. (%)	1/14 (7)	0/47 (0)	0.230
FEV1/FVC % predicted	100 [93–104]	100 [98–106]	0.375
<LLN—no. (%)	1/14 (7)	0/47 (0)	0.230
VA % predicted	103 [86–114]	96 [87–102]	0.245
<LLN—no. (%)	2/14 (14)	3/45 (7)	0.583
DLCO % predicted	102 [95–108]	95 [80–104]	0.085
<LLN—no. (%)	0/14 (0)	4/45 (9)	0.564
KCO % predicted	102 [91–114]	99 [86–112]	0.487
<LLN—no. (%)	0/14 (0)	3/45 (7)	1.000
TLC % predicted	105 [99–114]	95 [82–102]	0.005
<LLN—no. (%)	0/14 (0)	7/45 (15)	0.184

Data are reported as median [IQR] (continuous/numerical variables) or number (%) (binary/categorical variables). *p*-values are computed by the independent Mann–Whitney test (continuous variables) or Fisher test (binary variables). ** *n* = 4 and *n* = 7 missing data (CT- and CT+, respectively). Abbreviations: CRP = C-reactive protein, DLCO = diffusion capacity for carbon monoxide, FEV1 = forced expiratory volume in the first second, EQ5D = European quality of life index version 5D; FVC = forced vital capacity, HADS = Hospital Anxiety and Depression Scale, KCO = carbon monoxide transfer coefficient, LLN = lower limit of normal, mMRC = modified Medical Research Council, SGRQ = St. George’s Respiratory Questionnaire, TLC = total lung capacity, VA = alveolar volume, WBC = white blood cells.

## Data Availability

The datasets generated and analyzed during the current study are not publicly available due to individual privacy policy. However, clarifications are available from the corresponding author upon reasonable request.

## Abbreviations

COVID-19	coronavirus disease 2019
DLCO	diffusing capacity for carbon monoxide
FEV1	forced expiratory volume in the 1st second
FVC	vital capacity
GGO	ground-glass opacity
KCO	carbon monoxide transfer coefficient
mMRC	modified Medical Research Council
PFT	pulmonary function test
TLC	total lung capacity
VA	alveolar volume
